# A New Method of High-Precision Positioning for an Indoor Pseudolite without Using the Known Point Initialization

**DOI:** 10.3390/s18061977

**Published:** 2018-06-20

**Authors:** Yinzhi Zhao, Peng Zhang, Jiming Guo, Xin Li, Jinling Wang, Fei Yang, Xinzhe Wang

**Affiliations:** 1 School of Geodesy and Geomatics, Wuhan University, Wuhan 430079, China; yz_zhao_gnss@163.com (Y.Z.); coffeeyang@whu.edu.cn (F.Y.); wxz_gnss@foxmail.com (X.W.); 2 Key Laboratory of Precise Engineering and Industry Surveying of National Administration of Surveying, Mapping and Geoinformation, Wuhan University, Wuhan 430079, China; 3 Research Center for High Accuracy Location Awareness, Wuhan University 430079, China; 4 College of Geology Engineering and Geomatics, Chang’an University, Xi’an 710054, China; lixin2017@chd.edu.cn; 5 School of Surveying and Spatial Information Systems, The University of New South Wales, Sydney 2052, Australia; Jinling.wang@unsw.edu.au

**Keywords:** differential pseudolite system, pseudolite differential positioning, ambiguity function method, LAMBDA method

## Abstract

Due to the great influence of multipath effect, noise, clock and error on pseudorange, the carrier phase double difference equation is widely used in high-precision indoor pseudolite positioning. The initial position is determined mostly by the known point initialization (KPI) method, and then the ambiguities can be fixed with the LAMBDA method. In this paper, a new method without using the KPI to achieve high-precision indoor pseudolite positioning is proposed. The initial coordinates can be quickly obtained to meet the accuracy requirement of the indoor LAMBDA method. The detailed processes of the method follows: Aiming at the low-cost single-frequency pseudolite system, the static differential pseudolite system (DPL) method is used to obtain the low-accuracy positioning coordinates of the rover station quickly. Then, the ambiguity function method (AFM) is used to search for the coordinates in the corresponding epoch. The real coordinates obtained by AFM can meet the initial accuracy requirement of the LAMBDA method, so that the double difference carrier phase ambiguities can be correctly fixed. Following the above steps, high-precision indoor pseudolite positioning can be realized. Several experiments, including static and dynamic tests, are conducted to verify the feasibility of the new method. According to the results of the experiments, the initial coordinates with the accuracy of decimeter level through the DPL can be obtained. For the AFM part, both a one-meter search scope and two-centimeter or four-centimeter search steps are used to ensure the precision at the centimeter level and high search efficiency. After dealing with the problem of multiple peaks caused by the ambiguity cosine function, the coordinate information of the maximum ambiguity function value (AFV) is taken as the initial value of the LAMBDA, and the ambiguities can be fixed quickly. The new method provides accuracies at the centimeter level for dynamic experiments and at the millimeter level for static ones.

## 1. Introduction

The outdoor global navigation satellite system (GNSS) has been developed and widely applied, but it is difficult to obtain reliable GNSS signals in sheltered environments or indoors. A pseudolite system can make up for the shortage of GNSS in these environments [1,2]. However, there are also many problems in the indoor pseudolite systems, such as clock synchronization, multipath effect, noise, fixed satellite constellations, the high precision requirement of initial values, and so on. All of them are significant challenges for indoor pseudolite positioning.

At present, the most mature indoor pseudolite system is produced by LOCATA. A proprietary TimeLoc technology for tight time synchronization, allowing the clock difference in the carrier phase measurement equation to be ignored is utilized [3], which brings a large additional cost at the stage of system deployment. The clock synchronization error can be controlled within 30 ps in the LOCATA system. The horizontal error of the non-differential positioning is less than 6 cm, and the precision of the altitude direction is about 15 cm [4]. Kim et al. proposed a new concept of pseudolite-based navigation to overcome the receiver modification problem of the pseudolite-based positioning systems. The precision at the meter level can be obtained even in poor geometry tests [5]. Xu et al. built a new pseudolite-based indoor positioning system. The satellites clock can be synchronized with each other and signal can be processed by the standard receivers. High-accuracy horizontal positioning in both static and dynamic situations could be provided based on the simulation results [6]. Wan et al. used the simulated data of indoor pseudolites to verify the high positioning precision after the ambiguities are fixed through the LAMBDA method and the extended Kalman filter (EKF). Zhang et al. used carrier interferometry to conduct analyses on indoor pseudolite positioning [7]. Li et al. used the improved particle swarm algorithm to improve the search efficiency of AFM to achieve high precision positioning of a single epoch and the precision can reach the centimeter level [8]. Fujii et al. introduced a novel use of pseudolites to cope with pseudolites’ conventional problems. The results showed that the positioning accuracy varies from the centimeter- to the meter-level according to the geometric structure. They believed that the convergence precision of initial value was also important for positioning [9]. By using commercial pseudolites and independently-developed software receivers, an indoor pseudolite observation system was realized in our group. The fixed ambiguity resolution (AR) was obtained through the high accuracy carrier phase observations. The dynamic positioning precision can reach the centimeter level.

Considering the current research status, the key to achieve high-precision indoor pseudolite positioning is the use of carrier phase observations, and the core problem is how to fix the ambiguities correctly [10]. Generally speaking, the methods of AR can be divided into two categories. One is searching based on the coordinate domain. The integer nature of ambiguity is used to obtain the coordinates directly instead of solving the ambiguity explicitly in such methods [11]. The common method is the ambiguity function method (AFM) [12,13,14,15]. The other is searching in the ambiguity domain. The most popular method is LAMBDA [16,17,18]. This method is reliable and accurate, but a higher accuracy of initial values is needed in indoor pseudolite positioning [8]. On the acquisition of the initial values, due to the fixed constellation of the pseudolites, the static observations are almost the same. Most of the research institutes prefer to use KPI [19], including LOCATA. Nevertheless, it is difficult to obtain the known fixed point in a practical application, and it is not convenient to operate in dynamic observation. In addition, even if the known point has the initial precision at the decimeter level, the ratio of squared residuals of the best integer vector to the second-best vector is remarkably uncertain. The realization of initialization without high-accuracy known points has always been a vital research point in pseudolite positioning. To solve these problems, a new method is proposed in this paper. This method cannot only eliminate the limitation of KPI, but can also obtain the initial value satisfied with the high accuracy requirement of LAMBDA, so as to achieve high-precision indoor pseudolite positioning.

Focusing on a relatively cheap commercial pseudolite system, the double difference (DD) observation equation is utilized to eliminate or weaken the clock difference, phase center deviation, and so on. Firstly, the approximate coordinates of rover station can be obtained based on the differential pseudolite pseudorange method (DPL), and the Gauss-Newton iteration is used to improve the precision. Then, a search space of the AFM is established in the corresponding epoch and the coordinate of the highest AFV can be obtained. Moreover, the best step length and search scope are explored, and the wrong peaks of the AFM are eliminated by some constraint strategies. At present, the corresponding coordinates of the real peak can reach the centimeter level, which can meet the initial accuracy requirement of LAMBDA. Finally, the LAMBDA method is applied to fix the ambiguities of the DD carrier phase, and the iterative EKF is employed for parameter estimation. Thus, the high-precision positioning of indoor pseudolite is achieved.

## 2. Algorithm

**Figure 1:**
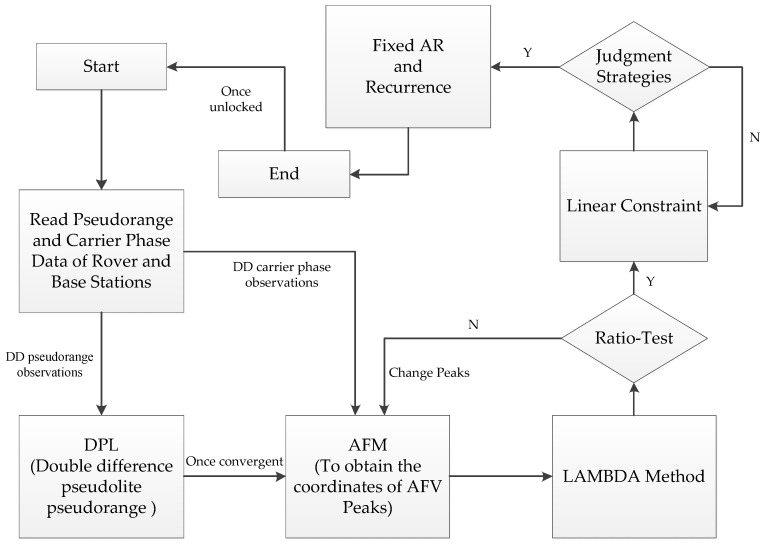
The procedure of the whole algorithm.

The indoor pseudolite system includes the following characteristics: first of all, the positioning error source, such as the satellite coordinate error, the satellite clock difference, the phase center deviation, multipath effect, and noise. Secondly, the constellation of the indoor pseudolite system is fixed, causing the static observations to be basically consistent without considering the noise. In view of the characteristics as above, there is a certain difference between the indoor pseudolite and the GNSS positioning. A new method is proposed in the paper and the procedure of the whole algorithm is shown in Figure 1.

### 2.1. Double Difference Observation Model in the Pseudolite System

Based on the above analyses of positioning error sources, the error of clock synchronization and phase center deviation can be eliminated or weakened by the DD model [20]. The DD observation equations [21,22,23] can be formulated as:

(1) $$\begin{array}{l} \nabla \Delta P_{br}^{ks}=\nabla \Delta \rho_{br}^{ks}+\nabla \Delta e_{br}^{ks}+\nabla \Delta M_{br}^{ks}+\nabla \Delta T_{br}^{ks}\\ \nabla \Delta \phi_{br}^{ks}=\nabla \Delta \rho_{br}^{ks}+\lambda \nabla \Delta N_{br}^{ks}+\nabla \Delta \varepsilon_{br}^{ks}+\nabla \Delta m_{br}^{ks}+\nabla \Delta T_{br}^{ks} \end{array}$$

where ∇Δ is a DD operator; *r* and *b* represent the rover and base stations, respectively; *k* and *s* represent pseudolites. The pseudorange and carrier phase observations are represented by *P* and *ϕ*; *ρ* represents the geometric distance of pseudolites to the receivers; *e* and *ε* denote the noise of the pseudorange and carrier phase observation, respectively; and *T* represents the tropospheric term. Considering the indoor environment, the height difference is small and the baseline is short, which causes the tropospheric term to have little effect on indoor positioning; *M* and *m* represent the multipath effects of pseudorange and carrier phase observation; *N* indicates the integer ambiguities of carrier phase observations; and *λ* indicates the wavelength.

From Equation (1), the structure of the pseudorange and carrier phase observation is similar, with the latter containing an ambiguous term. This structure implies that as long as the ambiguities can be resolved correctly, the carrier phase observation will be transformed to a high-accuracy receiver–pseudolite pseudorange [24].

### 2.2. Acquisition of Initial Coordinates by DPL

Thanks to the large number of GNSS satellites, excellent quality of signals, and stable and strictly synchronized clocks, it is easy to obtain the initial position using SPP. However, due to the constellation of pseudolites, multipath effect, noise, and the instability of the clocks, it is difficult to obtain the initial information by SPP in indoor positioning. As mentioned in the previous section, most of the research institutes prefer to use KPI to achieve high-precision positioning by the DD carrier phase model. However, it is difficult to obtain a fixed point in an engineering application, and it is not convenient to operate in the dynamic measurement. Therefore, we are going to eliminate the limitation of the KPI.

The DD pseudorange positioning model is used to eliminate or weaken the clock difference and other errors. Gauss-Newton iteration is employed for non-linear LSE (least square estimation) and the measurement equations can be written by a general non-linear vector function as [25]:

(2) y=h(x)+v

where *h(x)* is a measurement vector function of a parameters vector *x*. The equation can be extended by using Taylor series around an initial vector ***x*_0_** as:

(3) $$h(x)=h(x_{0})+B(x-x_{0})+\ldots$$

where *B* is a partial derivatives matrix of *h(x)* with respect to *x* at *x = x*_0_:

(4) $$B={\frac{\partial h(x)}{\partial x}|}_{x=x_{0}}$$

If the initial parameters are adequately near the true values and the second and further terms of the Taylor series can be ignored. The equation follows:

(5) $$y-h(x_{0})=B(x-x_{0})+v$$

By applying linear weighted LSE for Equation (5), the normal equation for non-linear LSE can be obtained:

(6) $$B^{T}PB(\hat{x}-x_{0})=B^{T}P(y-h(x_{0}))$$

The estimated unknown parameter vector can be obtained by:

(7) $$\hat{x}=x_{0}+{\left(B^{T}PB\right)}^{-1}B^{T}P(y-h(x_{0}))$$

Due to the initial parameters ***x*_0_** (default initial value is (0, 0)) are not near the true values, the estimated parameters can be iteratively improved like:

(8) $$\begin{array}{l} \overset{\wedge }{x_{0}}=x_{0}\\ \overset{\wedge }{x_{i+1}}=\overset{\wedge }{x_{i}}+{\left(B^{T}PB\right)}^{-1}B^{T}P(y-h\overset{\wedge }{(x_{i}})) \end{array}$$

If the iteration is converged, the final estimated parameters can be obtained as: $\overset{\wedge }{x}=\underset{i\to \infty }{\lim}\overset{\wedge }{x_{i}}$.

The iterated LSE is often called the Gauss-Newton method [25]. Note that such iterations are not always converged by the simple Gauss-Newton method [25]. In order to improve the correctness and efficiency of the estimation, the estimated parameters of previous epoch is passed to the later epoch as initial values in our pseudolite positioning system. Then continue to calculate until the difference of coordinates between the epochs is minimal. The initial approximate coordinates can reach the decimeter level through the above steps.

### 2.3. Refinement of the DPL Convergent Coordinates by AFM

When the initial coordinate bias (ICB) reaches the dm-level, the LAMBDA fails to obtain the correct ambiguity resolution (cannot pass the ratio validation) [8]. Consequently, the AFM is applied in this paper, and then the search grid is established based on the convergence epoch of DPL. When using AFM, the size of the search space is generally determined by the coordinate precision of the rover station [8] (DPL is around the decimeter level). The precision of the coordinates after searching can be considered to be dependent on the step length. The precision is relatively high when the step length is small. That is, the coordinates of the AFV [26] can reach the precision level of the step length. For example, if the step length is 2 cm, the initial coordinate precision can reach the centimeter level. The definition of AFM corresponding to the single-frequency carrier phase observations is given as follows:

(9) $$AFV(X_{c},Y_{c},Z_{c})=\frac{1}{n}\sum_{i=1}^{n}\cos2\pi (\phi_{real}^{}[E_{base}|E_{rover}]-\phi_{cal}^{}[E_{base}|X_{c},Y_{c},Z_{c}])$$

where *E_base_* is the base station. $\phi_{real}[E_{base}|E_{rover}]$ represents the DD observations of the base and rover stations. (*X_c_*, *Y_c_*, *Z_c_*) represents the coordinate candidate value in the search scope of the rover station. $\phi_{cal}^{}[E_{base}|X_{c},Y_{c},Z_{c}]$ represents the DD observations calculated from the candidate value of the rover station. Due to the integer nature of the ambiguity, it remains an integer after DD. If the coordinates of the rover station are found correctly, the cosine value of the difference should be 1. The noise error is always the key factor to the success rate of AFM [27]. Considering the effect of noise and multipath, the highest AFV is close to 1. Afterwards, the coordinate corresponding to the highest AFV is taken as the optimal solution of the initial value for the rover station.

However, there are two important defects in the traditional AFM method: efficiency and reliability [28]. The efficiency of AFM depends on the search space and the step length. The size of the search space is determined by the convergence precision of DPL in our method, and the step length will be explored in the experiments section. In addition, there is a multi-peak problem in AFM because the value is calculated by a cosine function. The reasonable values of the step length and the search space can also effectively reduce the wrong peaks. Additionally, the unreasonable expansion of the search space will inevitably lead to the emergence of the wrong peaks, which will also be described in the post. The decimeter level precision of the DPL is also a guarantee for the reliability of AFM. In order to further enhance the reliability of AFM, some strategies can also be added, such as a linear constraint which will be introduced later. 

### 2.4. LAMBDA Method

**Figure 2:**
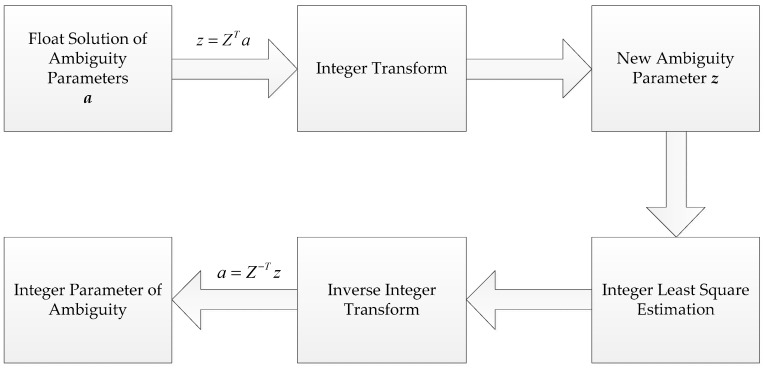
The process of fixing ambiguity by LAMBDA (Teunissen, 1995).

The initial coordinates of the centimeter level precision can be used to update the float solution. The LAMBDA method is used to search the ambiguities and the fixed solution is obtained through the ratio test. The LAMBDA method consists of two parts, including integer transform and integer least square estimation. The transform matrix Z is employed in integer transform and the float solution *a* is transformed into a new parameter vector *z*. *z* is easier to search than *a*. When the optimal integer estimation of *z* is obtained, it can be converted to an integer ambiguity solution through inverse transformation. The process is shown in Figure 2.

Some coordinates corresponding to wrong peaks can be excluded in the ratio test. If the wrong peaks still exist, some constraints can be used to solve this problem. For example, a linear constraint can be added to the rover station. The coordinate of the real peak will change along a straight line and then the wrong peaks will be excluded. The coordinate corresponding to the real peak here can reach the initial coordinate accuracy requirement of the LAMBDA method in the indoor pseudolite system.

### 2.5. Kalman Filter

The ambiguities between the epochs have close ties, so that the parameter estimation is usually carried out by using EKF in DD carrier phase positioning. Once the ambiguities are fixed correctly, the high-precision coordinate vector can be obtained. Although the LSE (least square estimation) is widely applied in the outdoor DGPS [29], we only use DPL for static initialization to obtain the outline coordinates. Here, the DD carrier phase positioning is taken as an example.

It can be considered that the coordinates remain the same because the rover station is a state of low dynamic or static in indoor positioning. The transition matrix is ***I*** and the covariance matrix of the system process noise is ***O***, but the measurement noise exists. The observation and the state model are as follows:

(10) $$\begin{array}{l} X_{k}=\Phi_{k-1}X_{k-1}+\omega_{k-1}\\ Z_{k}=H_{k}X_{k}+v_{k} \end{array}$$

where *k* is the current epoch; ***X*** represents the state vector, including the coordinate vector of the rover station and the ambiguities, that is, [***r*** ∇ΔN_q*q_]**^T^**; *q* depends on the number of DD observations. If the number of satellites is five, the corresponding *q* equals four; Φ is the transition matrix ***I***; *ω* is the dynamic process noise of the system; ***Z*** is a measurement vector after linearization; H is a design matrix [***I_r_*** *γ*]; and *v* indicates the measurement noise. If the dynamic process noise and the measurement noise of the system are Gaussian white noise, then the parameters have the following statistical characteristics:

(11) $$\begin{array}{l} E\left(\omega_{k}\right)=0,\;Cov\left(\omega_{k},\omega_{k}\right)=Q_{k}\\ E\left(v_{k}\right)=0,\;Cov\left(v_{k},v_{k}\right)=R_{k}\\ Cov\left(\omega_{k},v_{k}\right)=0 \end{array}$$

After the initial coordinate is obtained, the corresponding initial state parameter ***X*_0_** and its variance covariance matrix *P*_0_ can be given. The basic formula of EKF follows:

(12) $$\begin{array}{l} K_{k}=P_{k,k-1}H_{k}^{T}{\left(H_{k}P_{k,k-1}H_{k}^{T}+R_{k}\right)}^{-1}\\ P_{k,k-1}=\Phi_{k-1}P_{k-1}\Phi_{k-1}^{T}+Q_{k-1},\;P_{k}=\left(I-K_{k}H_{k}\right)P_{k,k-1}\\ X_{k,k-1}=\Phi_{k-1}X_{k-1},\;X_{k}=X_{k,k-1}+K_{k}V_{k}\\ V_{k}=Z_{k}-H_{k}X_{k,k-1} \end{array}$$

where ***K_k_*** is the Kalman gain matrix, and ***V_k_*** is the residual between the observations and predictions; ***X_k,k−1_*** is the predictive value, and ***P_k,k−1_*** is the predictive variance matrix.

When positioning, the iterative EKF is usually used to weaken the linearization error. The threshold of iteration is judged according to the residuals of the observations or the number of iterations is given directly. In order to avoid the problem of divergence caused by the imprecise noise of a given motion model during the Kalman filtering, the weight can be dynamically adjusted according to the residual matrix to achieve the robust Kalman filter [30].

As long as the pseudolites do not lose the lock, the positioning precision at the centimeter level in the dynamic measurement can be obtained. The cycle slips (CS) can be detected by LLI (loss of lock indicator) in the input measurement data. In order to avoid the wrong CS fix, if the CSs are frequent, or the signal is interrupted, the initial coordinate value can be searched again through the above algorithm, and the search space can be established on the basis of the interrupt value. For the sake of convenience, the improved algorithm is named DAFV-LAMBDA.

## 3. Experiment and Analyses

**Figure 3:**
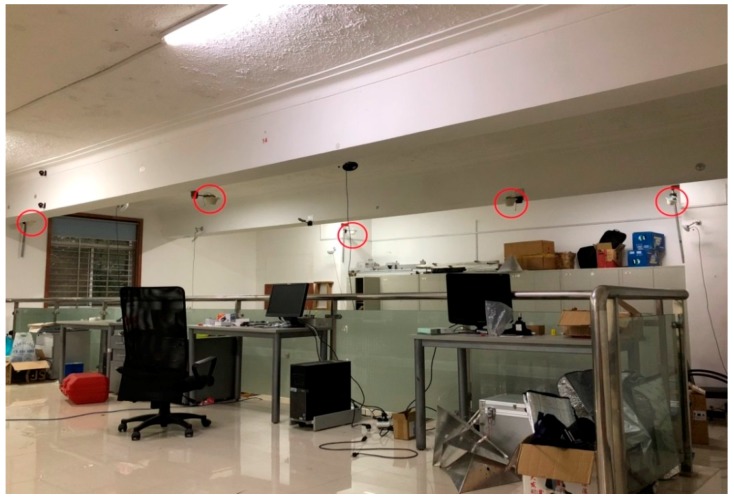
Distribution of pseudolite antennas.

GSG-L1 pseudolites have been used in the experiments, and five satellites have been set up in the laboratory. The ranging signals with C/A codes are transmitted on the GPS L1 frequency. The RF front end of the receiver is a USRP, and a full series of GNSS signals can be captured with a suitable sub-board. The pseudolite system is reloaded in the USRP with two DBSRX sub-plates, which can capture a signal of 800 MHz–2.4 GHz. Taking a certain mark as the origin point, the two horizontal directions of the ground are X and Y axis, respectively. The Z axis is perpendicular to the ground, and then the indoor independent coordinate system is established. The location of the pseudolites and some control points are accurately measured by a total station in the room. For convenience sake, all experiments are carried out on the plane (on the ground). In the program, the direction of Z is strongly constrained, and the precision of the plane coordinates is mainly explored. Additionally, the interval of data sampling is 0.1 s. The pseudolite positioning system is set up in a large laboratory (10 × 7 × 4 m^3^). Five pseudolites are placed on the ceiling of the room, as shown in Figure 3.

Three groups of experiments are carried out. The zero baseline static test is used to verify the feasibility of our system and an antenna is shared between the base station and rover station in the test. The short baseline static test is used to explore the effect and feasibility of each step in our algorithm and the precision of static positioning. The length of the baseline in the short baseline static test is about 1 m due to the small indoor space. Based on the results of static tests, the short baseline dynamic experiment is used to verify the positioning effect of DAFV-LAMBDA in dynamic environment and a special strategy to exclude incorrect peaks is also applied, which has been mentioned in Section 2.4. The length of the baseline changes from 0.5 m to several meters in the short baseline dynamic experiment.

### 3.1. Analyses of Static Experiments

#### 3.1.1. Accuracy Analyses of Observations

**Figure 4:**
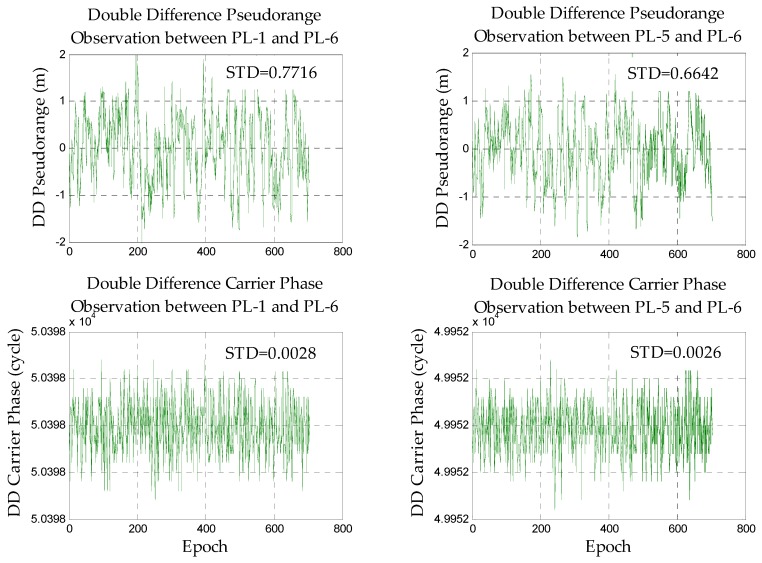
The double difference observations in the zero baseline.

**Figure 5:**
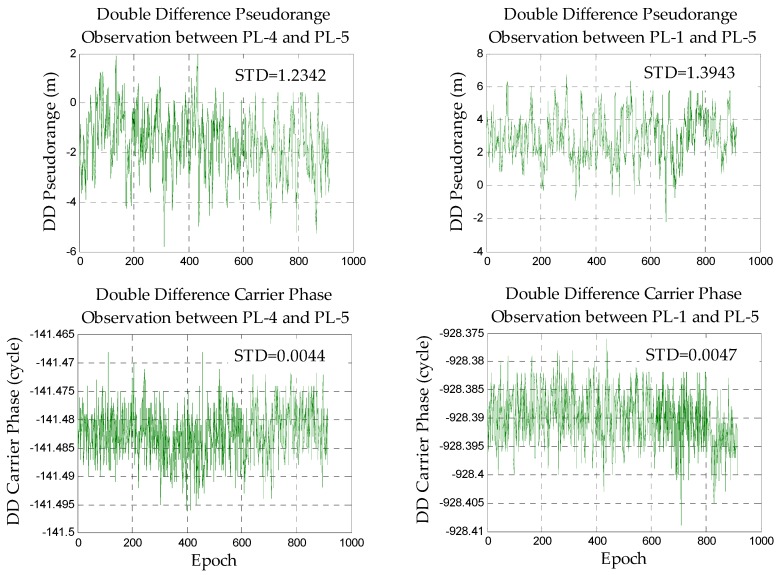
The double difference observations in the short baseline.

First, the accuracy of the observation data is analyzed to ensure reliable data, and the main error sources that affect the positioning can be explored. Due to the complexity of the indoor environment, this paper mainly focuses on the static DD pseudorange/carrier phase observations of the zero baseline and short baseline (clock error and antenna phase deviation should be eliminated or weakened by DD). In the case of the zero baseline, it can be considered that the multi-path effect of the rover station and base station is basically the same. The main error which affects positioning is noise. With respect to the short baseline, the impact of the rover and base station is different from each other, so the magnitude of error caused by several positioning error sources can be explored. The static DD pseudorange/carrier phase observations of the zero baseline and short baseline are shown in Figure 4 and Figure 5.

It can be found that the standard deviation (STD) of the carrier phase observations is very small and stable after DD, and its internal accuracy is high. It shows that if the ambiguities are fixed correctly, the carrier phase observations can be used to achieve high-precision positioning. The overall fluctuation of the DD pseudorange observations is also relatively stable. It indicates the feasibility of positioning. However, its STD reaches the meter level which means that we cannot directly apply it to high-precision positioning (centimeter level). At the same time, some errors still exist in part of the DD pseudorange observations and the weight should be adjusted according to the DD residual.

Comparing the data of the zero baseline experiment with the short baseline experiment, we can find that the quality of the short baseline observations is far less than the zero baseline due to the very large impact of the multipath and noise in the indoor environment.

#### 3.1.2. Results of Pseudolite Differential Pseudorange Positioning

Based on the analyses of the previous section, the static differential pseudorange positioning experiments of the short baseline are performed, respectively.

In the outdoor GNSS differential pseudorange positioning, the initial value of the rover station can be given by SPP because the constellations of satellites are changing at all times and the number of satellites is redundant. Thus, a more accurate value can be obtained in a few epochs. Based on the previous analyses, SPP cannot be carried out in indoor pseudolite system. The DPL method is used and the initial value of the rover station is arbitrarily given in this paper. For example, the plane coordinates of the initial value are taken as (0, 0) and (4, 4) in the experiments. Therefore, the rover station does not need to initialize on the known point

**Figure 6:**
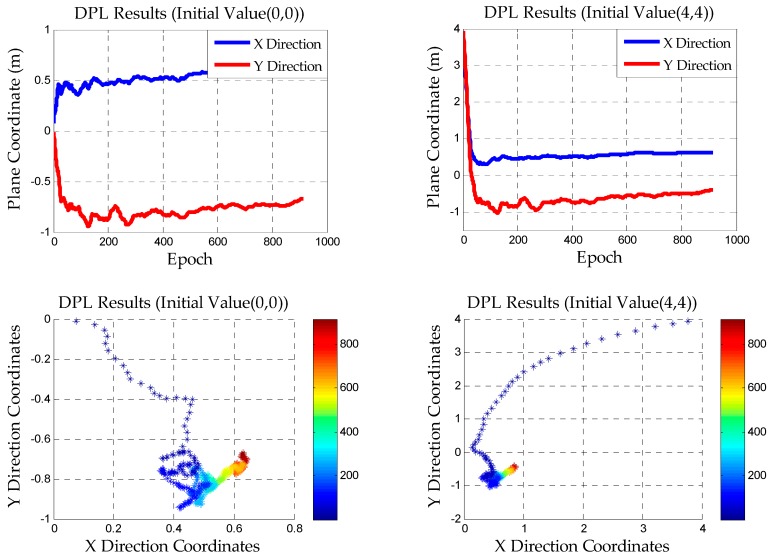
The results of DPL in the short baseline static experiment.

After using the strategies described in the previous chapter, Figure 6 reveals the results of DD pseudorange positioning.

The plane scatter graph in the lower part of Figure 6 is used to demonstrate the convergence process, which can intuitively reflect the speed and the final convergence accuracy. It can be found that the initial coordinate of decimeter precision can be obtained in a short time by the DPL method. It is not necessary to wait for complete convergence to obtain the coordinates. Thus, the feasibility of indoor pseudolite positioning without using the KPI method can be explained. That is to say, we can obtain the positioning precision at the decimeter level in the first few epochs and the initial value is also arbitrarily given. This is of great significance to the use of AFM in the next chapter. In addition, the short baseline static experiment is a preview of the initialization process in the dynamic test.

Indoor pseudolite dynamic experiments are generally based on short baselines. However, the multipath and noise have too much of an effect on the pseudorange observations of the rover station under the dynamic condition. If the DPL is used directly, the result will be poor. Therefore, it is impossible to use DPL for dynamic positioning, but it is only employed for static initialization to eliminate the limitation of KPI.

#### 3.1.3. Results of AFM and the Exploration of Efficiency and Reliability

Based on the convergence value of DPL, the search grid of AFM is established. The coordinates of the highest AFV (the precision depends on the search step length) can meet the initial value requirement of the LAMBDA method.

Since the sub-meter precision can be obtained by static DPL, the search space of AFM can be adopted at about 1 m. This will play an important role in the study of efficiency and the multiple peaks problem in the following processing.

(1) The Effect of AFM Step Length and Scope on Multi-Peaks and Efficiency

Step length is one of the factors that affect the efficiency and reliability of AFM. The core idea of this method is to obtain an initial coordinate that meets the requirement of LAMBDA through AFM, so the step length to be taken does not need to be too small (the centimeter level is enough).

Search efficiency does not depend on the experimental data itself. In this paper, the short baseline static experiment is taken as an example to explore the search efficiency under different conditions. The statistical results are as follows:

In Table 1, it is known that if the search range is larger and the search step length is smaller, the efficiency will be greatly reduced. Therefore, the search scope should be reduced and the step length should be increased properly, so that the initial coordinates to meet the accuracy requirement of LAMBDA method can be obtained in a very short time. Since the decimeter level precision coordinates can be obtained through the DPL method, the range can be 1 m. The step length can be 2 cm or 4 cm to ensure the precision of the coordinates correspond to the highest AFV.

The first step of the algorithm is static initialization. The initial coordinates of positioning are obtained through DPL + AFM and the LAMBDA method is still used in the following steps to obtain the correct ambiguity resolution (AR). Thus, the search time is calculated in the initialization stage (DPL + AFM) rather than in the positioning stage.

**Figure 7:**
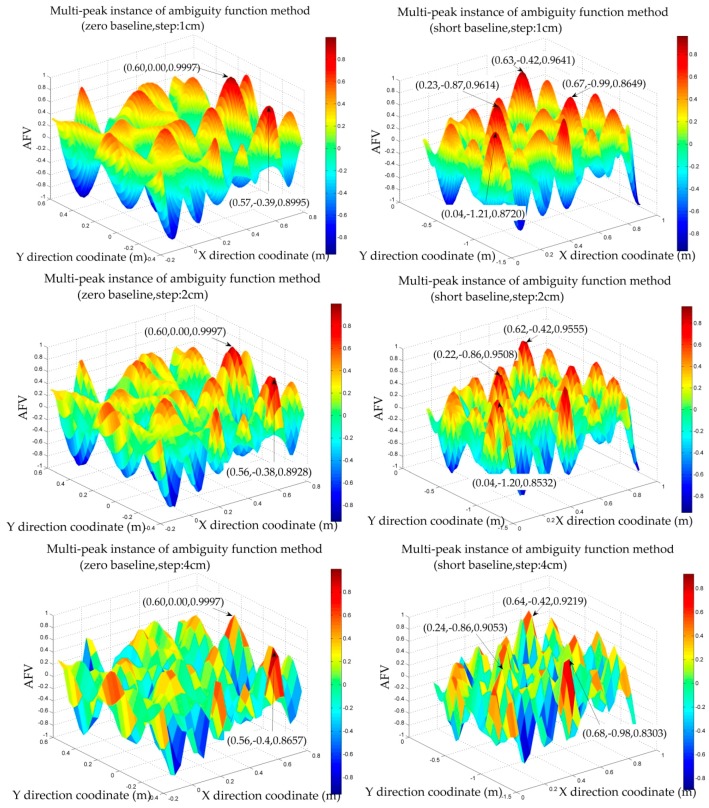
The effect of AFM step length on multi-peaks.

Figure 7 reveals the AFVs (ambiguity function values) whose search space is established based on the DPL convergence value in the zero and short baseline static experiments under different step lengths. The left side is the zero baseline static experiment. The step length is 1 cm, 2 cm, 4 cm, and the search space is 1 m. The right shows the short baseline static experiment. The step length is 1 cm, 2 cm, 4 cm, and the search space is 1 m, too.

It is intuitively shown that the AFVs near the peaks can be diluted when the step length is slightly larger. At the same time, the efficiency can be also improved, so as to achieve rapid initialization. Additionally, the precision of the coordinates is determined by the step length, but the step length should not be too large, otherwise it may hide the real peak. The wrong peak in the experiment will be explained in the next chapter.

The search scope is another factor affecting the efficiency of AFM, and it also affects the reliability of AFM [31,32]. The search scope in this paper is based on the convergence precision of DPL. Under the condition of ensuring a reasonable scope, a proper reduction in the search range can also be a good way to remove the wrong peaks.

(2) AFM Multi-Peaks and The Coordinate Precision Corresponding to the Highest AFV

If the parameters of AFM are not reasonable, it is difficult to avoid the emergence of the multi-peaks problem. We search in different epochs, respectively, to remove some wrong peaks, and the real peak should be consistent theoretically in different epochs. The other way to remove wrong peaks will be introduced in the dynamic experiment.

As to the initial accuracy of LAMBDA method in the indoor pseudolite system, it has been explained in detail in the literature [8], whose conclusion can be directly referenced. That is, the LAMBDA method needs the initial value at the centimeter level of precision. In this paper, the precision of the coordinates corresponding to AFVs will be compared with the exact coordinates.

In the zero baseline static experiment, all the coordinates corresponding to the large AFVs are shown in Table 2. In the experiment, the exact coordinates of the rover station are, in accordance with the base station, about (0.60, 0.00).

According to Table 2, we can find that all the coordinates corresponding to the larger AFVs in the zero baseline static experiment are nearly exact. Moreover, the coordinates corresponding to the highest AFV are close to the exact coordinates. The zero baseline experiment fully reflects that the proper expansion of the step length can effectively reduce the error coordinates near the real peak. It also verifies that if the step length is too large, it may cover the real peak.

In the short baseline static experiment, all the coordinates corresponding to the larger AFVs are shown in Table 3. In the experiment, the exact coordinate of the rover station is about (0.628, −0.424).

Since AFM is a mathematical operation of the DD observations, the quality of the data directly affects the conspicuousness of the peak. Therefore, the highest AFV obtained in the zero baseline experiment is more remarkable than the short baseline experiment.

According to Table 3, the coordinates corresponding to the highest AFV are less different from the exact coordinates. If we increase the step length appropriately, the larger AFVs will decrease, which is similar to the zero test. We have reason to draw the conclusion that the precision can reach the centimeter level and meet the initial requirement of the LAMBDA method. There are also some wrong peaks that exist even if we increase the step length and reduce the search space appropriately. Some other strategies will be introduced in the dynamic test.

#### 3.1.4. Results of the Static Experiments after the Ambiguities are Fixed

**Figure 8:**
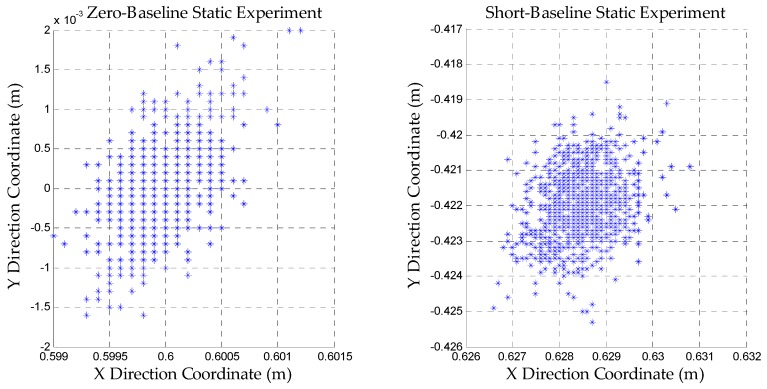
Results of static experiments after the ambiguities are fixed.

Figure 8 shows the results of static experiments after the ambiguities are fixed correctly. The reason why the zero baseline experiment is performed is that our pseudolite system should be proved to be intact and the data quality is good without considering the interference from the external environment. The short baseline experiment is used to verify the feasibility of the dynamic positioning initialization.

According to Figure 8, it can be found that the precision of the positioning can reach the millimeter level. This illustrates the feasibility of our algorithm. The ambiguities can be fixed to achieve high precision positioning.

### 3.2. Analyses of the Dynamic Experiment

#### 3.2.1. The Convergence Results of Static Initialization and Large Peaks of AFM

A dynamic test is carried out on a fixed line rail so that the dolly (rover station) can be controlled. Static initialization is also the first step in the dynamic experiment. The reference antenna remained stationary throughout data collection. The coordinates of the starting point are about (−0.686, −0.525). DPL is also used and the convergence coordinates is about (−0.66, −0.58). The search space of AFM is set up according to the coordinates (−0.66, −0.58). The search scope is 1 m and the step is 2 cm.

Table 4 shows the results of large AFVs. The coordinate corresponding to the largest one is about (−0.68, −0.52) and there are some wrong peaks that exist. Some strategies will be used to remove the wrong peaks. Although the AFV of the real peak is the largest in the experiment, some relatively large AFVs are also taken as wrong peaks for analysis in order to increase the robustness of the algorithm.

Reducing the search scope and increasing step length is a common method to exclude the wrong peaks of AFM. Some constraint methods are used in the test and the only real peak can be identified effectively. In the next section, the linear constraint will be introduced.

#### 3.2.2. The Linear Constraint to Recognize the Real Peak

The linear constraint used in this section can be divided into two parts. In one of the experiments, there is a straight track. The rover station can be controlled to move along the rail for a short distance. Only the coordinate of the real peak will change along a straight line, which is mentioned in Section 2.4. In the other one, a dolly is taken as a carrier instead of the fixed rail. However, the method is the same as the former.

**Figure 9:**
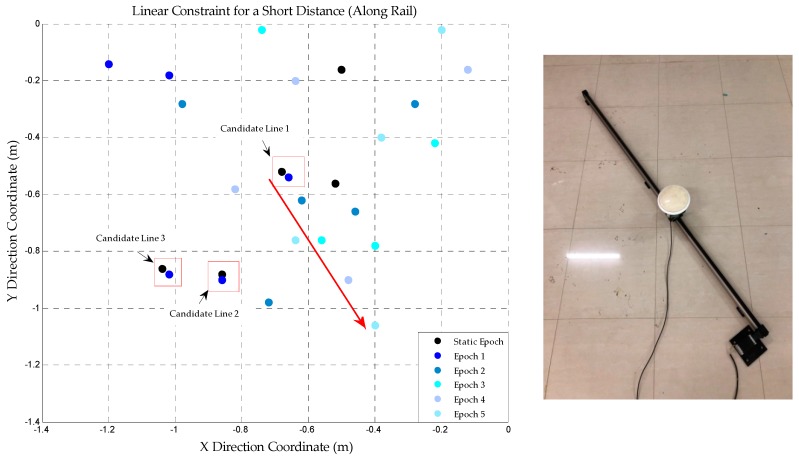
The trajectory obtained by AFM during the linear constraint (along rail), the right image shows the fixed rail for linear constraint test

After static initialization, the rover station is controlled to move along the straight rail for a short distance. AFM is applied during the dynamic perturbation dataset and a series of coordinates corresponding to the large AFVs can be obtained (including the wrong peaks). The correct trajectory should be a beeline so that the real peak can be found through this constraint strategy. The coordinates of the large AFVs are shown in Figure 9.

Figure 9 shows the results of AFM from static to dynamic linear constraint stage. The scatter is drawn every five epochs (interval: 0.1 s) in order to make the graph more clear and intuitive. According to Figure 9, only one trajectory is always in line with the characteristics of the linear constraint and the other coordinates are all discrete distribution. It indicates that the real peak is uniquely identified. In addition, the right picture of Figure 9 is the actual photograph of the fixed rail and motion carrier (rover station).

The first coordinate in correct trajectory is the coordinate corresponding to real peak at static initialization stage. In the program, the coordinates of static epoch and the first epoch of dynamic linear constraint should be close. The candidate lines could be found and the slopes of lines would be obtained. The coordinates of the first and second epochs can also form a straight line, whose slopes should be similar to the former (the first epoch and static epoch). Therefore, the right trajectory can be found by setting the threshold of the slopes difference. Once the real peak is determined, the corresponding coordinate can be used as the initial value of LAMBDA, so as to achieve high accuracy dynamic indoor positioning.

**Figure 10:**
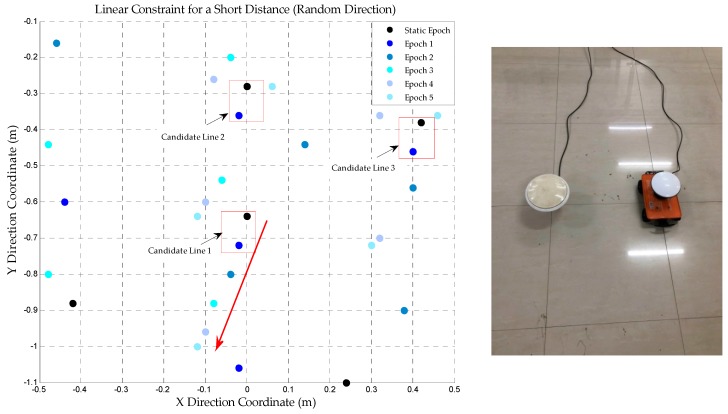
The trajectory obtained by AFM during the linear constraint (random direction), the right image shows the dolly for linear constraint test

In order to verify the reliability of the linear constraint to remove the wrong peaks another test is conducted. In this test, the dolly is not fixed on the track; instead, it can be controlled to move in any directions. We put the antenna on the top of the dolly and control it to move about 30 cm in a certain direction. AFM is also used to obtain the coordinates of each epoch and the result is shown in Figure 10.

According to Figure 10, only one trajectory is always in line with actual linear motion. The real peak can be identified by the method described in the preceding paragraph. It reveals that, instead of using the fixed rail, the wrong peaks can be also removed with a similar approach in practical applications and only a short distance linear constraint is needed. The right side of Figure 10 is the actual photograph of the base and rover station whose antenna is placed on a radio-controlled dolly.

#### 3.2.3. The Positioning Results of DAFV-LAMBDA in the Dynamic Test

**Figure 11:**
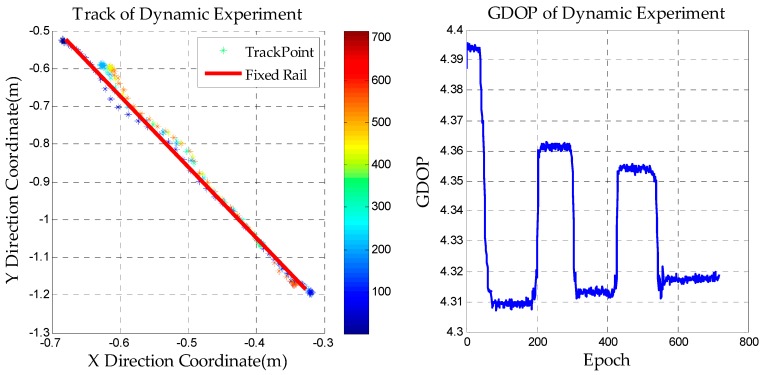
The results of the dynamic experiment.

We install the receiver on the dolly and let it move back and forth on a fixed straight line track. After static initialization, the AFM search space is set up based on the DPL convergence value. When the real peak is obtained, its coordinate is used as the initial value to update the float solution. Then the LAMBDA method is used to fix the ambiguities and dynamic positioning can be achieved. The result is shown in Figure 11.

After the ambiguities are fixed, the results conform to the actual movement and the maximum deviation of the positioning is not more than 5 cm. The picture on the right reflects the change of the GDOP value in the dynamic experiment, and the GDOP is widely used to reflect the location of the layout of the pseudolites. In the experiment, the GDOP value in the moving area of the dolly is less than 5, which indicates that the location of the pseudolites in the experiment is reasonable. It also shows the special DOP value caused by the fixed constellation of the pseudolite system.

Combining all the experimental results, we can consider that the static positioning precision can reach the millimeter level and the dynamic positioning can reach the centimeter level. This shows that, using this method, high-precision indoor pseudolite positioning can be realized. We can also overcome the limitation of KPI and the high accuracy requirement of LAMBDA for initial value.

## 4. Conclusions

For the problems existing in the use of KPI in indoor pseudolite positioning, we propose a method that does not need to initialize on known points and also meets the initial accuracy requirement of the LAMBDA method, so as to achieve high-precision positioning, named DAFV-LAMBDA. In the DPL portion, the feasibility of the static DD pseudorange positioning and the unreliability of the dynamic DD pseudorange positioning are analyzed. Moreover, the precision and the speed of convergence have been improved to some extent and the initial coordinates at the decimeter level can still be obtained without the need for known information. In the AFM portion, it is believed that the convergence precision of DPL affects the size of the search scope. Additionally, some methods to deal with the problem of multiple peaks are also provided, for example, applying a linear constraint. At the same time, some sections focus on the step length of AFM, and the efficiency is analyzed. Finally, we draw the conclusion that the coordinate corresponding to the highest AFV can satisfy the precision demand of the LAMBDA method and the efficiency is high. Static and dynamic high-precision positioning can be achieved after the ambiguities are fixed. This method is a good method to eliminate the limitations of KPI and to overcome the weakness of the high-precision requirement of LAMBDA for initial values in indoor pseudolite positioning.

If there are still some incorrect peaks after using the multi-peaks processing strategy presented in this paper, we should propose more methods in the next step of the study. However, it is difficult to solve the multi-peaks problem perfectly in theory. It is still an open question. In addition, if the exact coordinates are needed, the static positioning precision can be further optimized by SNR constraints. It is also possible to increase the success rate of ambiguity through a partial ambiguity algorithm. Additionally, how to choose the threshold of the LAMBDA ratio test and improve the accuracy of pseudolite coordinates are also difficult to dispose. All of these are further research goals.

## Figures and Tables

**Table 1:** Search time under different conditions/seconds.

Search Scope/m	Search Step/cm				
	0.1	0.5	1	2	4
0.5	5.032	0.203	0.051	0.012	0.004
1	20.495	0.823	0.207	0.051	0.012
2	83.901	3.299	0.834	0.205	0.053

**Table 2:** The results of 1 m search scope under different step lengths (zero baseline).

**Search Step of 1 cm**		
**X Coordinate**	**Y Coordinate**	**AFV**
0.6	0	0.9997
0.6	−0.01	0.9926
0.6	0.01	0.9906
0.61	−0.01	0.9771
0.59	0.01	0.9767
0.59	0	0.9739
0.61	0	0.9724
0.6	−0.02	0.9693
0.61	−0.02	0.9657
0.6	0.02	0.9657
0.59	0.02	0.9636
**Search Step of 2 cm**		
**X Coordinate**	**Y Coordinate**	**AFV**
0.6	0	0.9997
0.6	−0.02	0.9693
0.6	0.02	0.9657
0.62	−0.02	0.9104
0.58	0.02	0.9097
0.58	0	0.8970
**Search Step of 4 cm**		
**X Coordinate**	**Y Coordinate**	**AFV**
0.6	0	0.9997
0.6	−0.04	0.8767

**Table 3:** The results of 1 m search scope under different step lengths (short baseline).

**Search Step of 1 cm**		
**X Coordinate**	**Y Coordinate**	**AFV**
0.63	−0.42	0.9641
0.23	−0.87	0.9614
0.23	−0.86	0.9607
0.63	−0.43	0.9602
0.62	−0.42	0.9555
0.22	−0.86	0.9508
**Search Step of 2 cm**		
**X Coordinate**	**Y Coordinate**	**AFV**
0.62	−0.42	0.9555
0.22	−0.86	0.9508
**Search Step of 4 cm**		
**X Coordinate**	**Y Coordinate**	**AFV**
0.64	−0.42	0.9219

**Table 4:** The large AFVs of the dynamic experiment (static initialization stage).

X Coordinate	Y Coordinate	AFV	remark	X Coordinate	Y Coordinate	AFV
−0.68	−0.52	0.9364	Real Peak	−0.52	−0.56	0.8957
−0.50	−0.16	0.9147	Wrong Peak	−0.86	−0.88	0.8916
−0.50	−0.14	0.9138	Wrong Peak	−1.04	−0.16	0.8831
−1.04	−0.86	0.9069	Wrong Peak	−0.34	−0.18	0.8599
